# Dcc Regulates Asymmetric Outgrowth of Forebrain Neurons in Zebrafish

**DOI:** 10.1371/journal.pone.0036516

**Published:** 2012-05-14

**Authors:** Jingxia Gao, Changwen Zhang, Bin Yang, Liu Sun, Cuizhen Zhang, Monte Westerfield, Gang Peng

**Affiliations:** 1 Institutes of Brain Science and State Key Laboratory of Medical Neurobiology, Fudan University, Shanghai, China; 2 Institute of Neuroscience, University of Oregon, Eugene, Oregon, United States of America; Harvard University, United States of America

## Abstract

The guidance receptor DCC (deleted in colorectal cancer) ortholog UNC-40 regulates neuronal asymmetry development in *Caenorhabditis elegans*, but it is not known whether DCC plays a role in the specification of neuronal polarity in vertebrates. To examine the roles of DCC in neuronal asymmetry regulation in vertebrates, we studied zebrafish anterior dorsal telencephalon (ADt) neuronal axons. We generated transgenic zebrafish animals expressing the photo-convertible fluorescent protein Kaede in ADt neurons and then photo-converted Kaede to label specifically the ADt neuron axons. We found that ADt axons normally project ventrally. Knock down of Dcc function by injecting antisense morpholino oligonucleotides caused the ADt neurons to project axons dorsally. To examine the axon projection pattern of individual ADt neurons, we labeled single ADt neurons using a forebrain-specific promoter to drive fluorescent protein expression. We found that individual ADt neurons projected axons dorsally or formed multiple processes after morpholino knock down of Dcc function. We further found that knock down of the Dcc ligand, Netrin1, also caused ADt neurons to project axons dorsally. Knockdown of Neogenin1, a guidance receptor closely related to Dcc, enhanced the formation of aberrant dorsal axons in embryos injected with Dcc morpholino. These experiments provide the first evidence that Dcc regulates polarized axon initiation and asymmetric outgrowth of forebrain neurons in vertebrates.

## Introduction

Neurons are polarized cells. The acquisition of correct polarity is a critical step in neuronal differentiation and subsequent circuit formation. Intracellular kinases and polarity proteins regulate cytoskeletal dynamics and intrinsic cellular programs that determine neuronal polarity [1]–[8]. In vivo, developing neurons encounter extracellular cues that promote asymmetric growth and direct the formation of correct polarity [9]–[14]. In *Caenorhabditis elegans*, the guidance receptor UNC-40 (DCC, deleted in colorectal cancer) is localized to the ventral side of the hermaphrodite specific neurons (HSN) before they extend axons ventrally towards the ventral nerve cord, where the UNC-6 (netrin) guidance cue is secreted [11]. Mutations in UNC-40 may result in randomly oriented neuronal processes. In *unc-40* loss of function mutants or in *unc-40* missense plus *unc-6* double mutants, HSN axons extend anteriorly, posteriorly, or dorsally, instead of towards the correct ventral position [15]. These studies suggest that the guidance receptor DCC is a key regulator of neuronal asymmetry in *C. elegans*. The functions of DCC in axon guidance and cell migration are conserved in bilateria [16]–[19], but few studies have examined the roles of DCC in neuronal polarity regulation in vertebrates.

We studied Dcc function in the zebrafish forebrain and found that it regulates asymmetric neuronal growth. Utilizing transgenic animals expressing fluorescent proteins, we determined that anterior dorsal telencephalic (ADt) neurons project axons ventrally. ADt neurons express Dcc before the axons emerge, and the Dcc ligand, Netrin1, is expressed in ventrally located cells. We found that knock down of Dcc function by antisense morpholino oligonucleotides causes ADt neurons to project aberrant axons dorsally. We further examined the axon projection patterns of individual ADt neurons and found that ADt neurons project axons dorsally or form multiple processes after knockdown of Dcc function. In addition, we found that knock down of the Dcc ligand, Netrin1, also caused the ADt neurons to project axons dorsally. Knockdown of Neogenin1, a guidance receptor closely related to Dcc, enhanced formation of aberrant dorsal axons in embryos injected with Dcc morpholino. These data show that Dcc regulates polarized axon initiation and asymmetric outgrowth of neurons in a vertebrate forebrain.

## Results

### ADt Neurons Project Axons Ventrally

**Figure 1:**
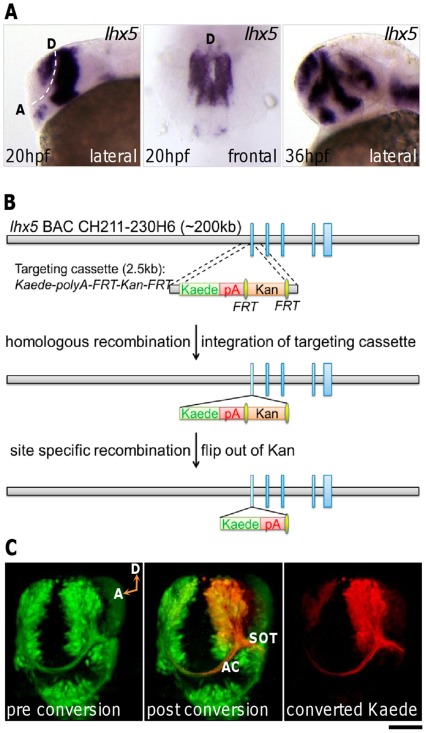
ADt neurons project axons ventrally. In this and subsequent figures, the probes used for whole-mount in situ hybridization are listed in the upper right corner of each panel. Developmental stages are indicated in the lower left corners. Lateral, animals mounted in lateral view, rostral to the left; Frontal, animals mounted in frontal view, dorsal to the top. (**A**) *lhx5* is expressed in the anterior dorsal region of the telencephalon. Dashed line marks the telencephalon-diencephalon border. D: dorsal; A: anterior ventral. Scale bar: 100 µm for lateral view; 60 µm for frontal view. (**B**) BAC modification via recombination methods. Vertical blue bars represent exons of the *lhx5* gene. The Kaede expression cassette replaced the first exon of *lhx5* gene. pA: polyadenylation signal sequence; Kan: kanamycin resistant marker; *FRT*, flippase recognition target. (**C**) Photo-conversion of Kaede in *Tg(lhx5BAC:Kaede)* transgenic embryos demonstrates ADt neurons project axons ventrally into the AC and SOT. A live *Tg(lhx5BAC:Kaede)* transgenic animal was mounted in tilted frontal view to reveal the AC and SOT simultaneously. The region of the left telencephalon was selected for photoconversion. D: dorsal; A: anterior ventral; AC: anterior commissure; SOT: supraoptic tract. Scale bar: 60 µm.

In the developing zebrafish forebrain, a cluster of cells are specified as neurons in the dorsal rostral region of the neural tube. By the pharyngula period, these telencephalic neurons have extended axons along a commissural tract (the anterior commissure, AC) and a descending tract (the supraoptic tract, SOT) [20]. In the anterior dorsal telencephalon, ADt neurons express *lhx5* (Fig. 1A), which encodes a LIM homeodomain protein that regulates the antagonism of Wnt signaling, thus promoting forebrain development [21]. To visualize the *lhx5* expressing ADt neurons and their axons in live intact animals, we generated transgenic zebrafish expressing the photo-convertible fluorescent protein Kaede [22] using a bacteria artificial chromosome (BAC) homologous recombination method [23], [24]. The BAC clone carried approximately 200 kb of zebrafish genomic sequence, of which approximately 110 kb was upstream of the transcriptional start site of the *lhx5* gene (Fig. 1B). The modified BAC construct contained Kaede coding sequence that replaced the first exon of the *lhx5* gene. Two lines of *Tg(lhx5BAC:Kaede)* stable transgenic fish were obtained after screening 80 of the modified BAC injected fish. The expression patterns of the Kaede protein were identical between the two lines. We maintained one of the *Tg(lhx5BAC:Kaede)* lines (referred to as *lhx5:Kaede* hereafter).

By the mid-pharyngula period at 36 hpf, fluorescent signal from transgenic Kaede expression was observed in the somata of the ADt neurons and axon tracts in the forebrain (Fig. 1C). To ascertain which axon tracts originated from the ADt neurons, we photo-converted Kaede protein in the ADt neuronal somata from the green light emission form to the red light emission form. Diffusion of the photo-converted, red colored Kaede from the ADt somata into the axons showed that the ADt neurons project axons ventrally into the AC and the SOT (Fig. 1C).

### ADt Neurons Express *dcc*

**Figure 2:**
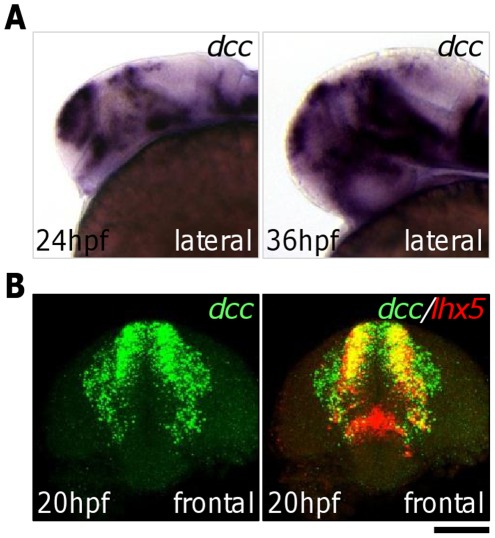
Expression patterns of *dcc* and *netrin*. (**A**) *dcc* is expressed in the dorsal telencephalic region at 24 hpf and 36 hpf. (**B**) *dcc* and *lhx5* are co-expressed in the dorsal telencephalon at 20 hpf. ADt neurons are migrating from their medial positions in the neural tube to lateral positions at 20 hpf. Scale bar: 100 µm for lateral view; 60 µm for frontal view.

The guidance receptor Dcc and its ligand Netrin1 are expressed in the central nervous system during zebrafish embryonic development [25]–[28]. During the pharyngula period (at 24 hpf and 36 hpf), *dcc* is expressed in dorsal telencephalic regions (Fig. 2A). Double labeling by fluorescent in situ hybridization demonstrated that *dcc* is co-expressed with *lhx5* in the ADt neurons at 20 hpf, a stage when the *lhx5:Kaede* labeled ADt neurons are still migrating and lack axonal processes (Fig. 2B). During these periods of development, *netrin1a* and *netrin1b*, the duplicated genes orthologous to the mammalian *Netrin1* gene, are expressed in the ventral midline region of the forebrain and ventral regions along the SOT ([27], [28], and data not shown). These expression patterns indicate that the guidance receptor Dcc is expressed in the right place at the right time to direct the asymmetric growth of the ADt neurons.

### DCC is Required For Correct Asymmetric Growth of the ADt Neurons

**Figure 3:**
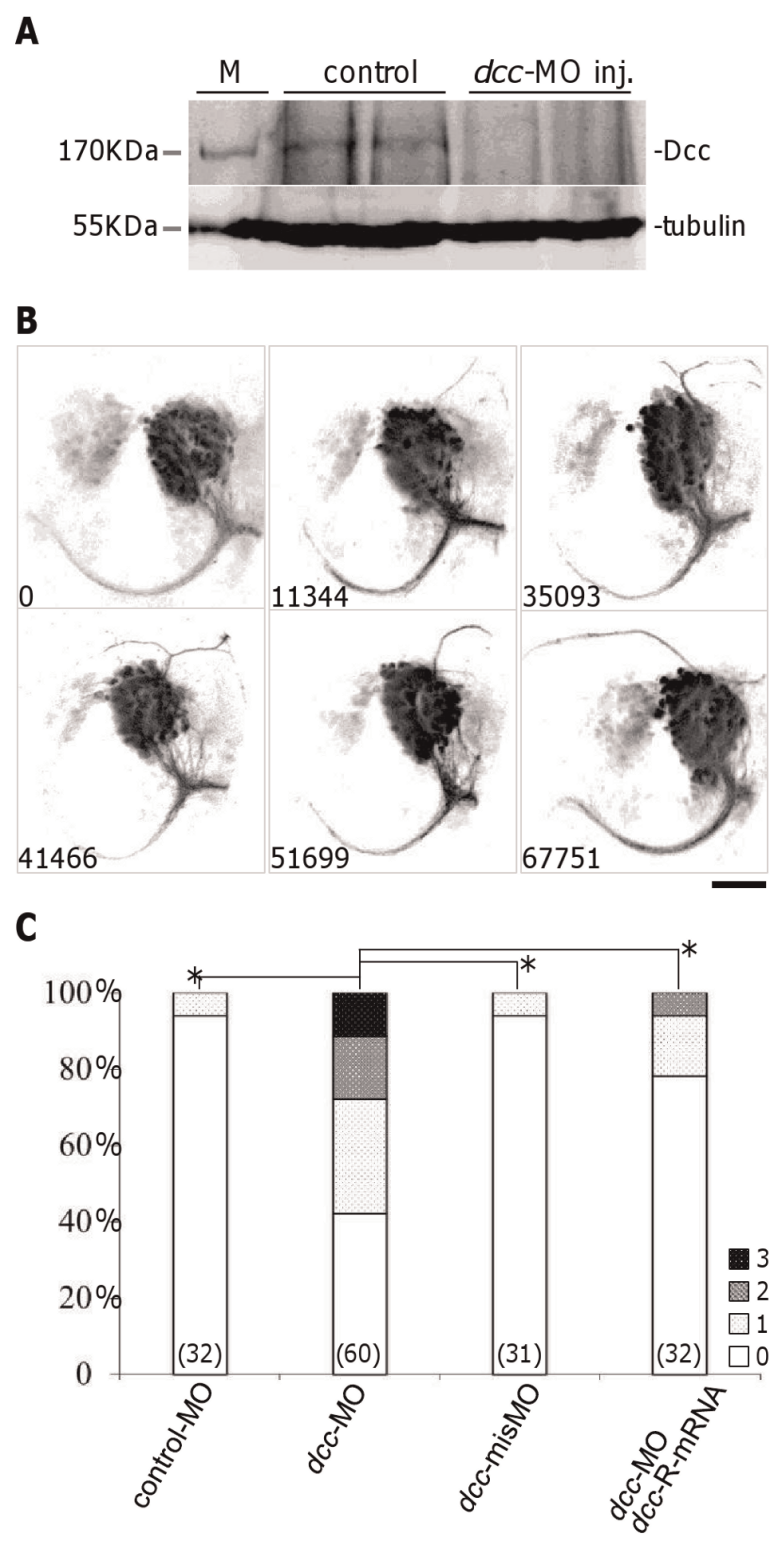
Dcc is required for correct asymmetric outgrowth of ADt neuronal axons. (**A**) Dcc expression was reduced after injection of *dcc* translation-blocking morpholinos into zebrafish embryos. Endogenous Dcc protein was detected as a band of approximately 170 kb. Tubulin served as a loading control. M: size marker. (**B**) ADt neurons project axons dorsally when Dcc function is inhibited by morpholino injection. Images of live animals were acquired as in Fig. 1C. The pixel intensity value of aberrant axon is shown in the bottom left corner of each panel. Scale bar = 50 µm. (**C**) Quantitation of ADt neuronal axon defects. Horizontal axis shows the treatment group labels and vertical axis shows the percentage of embryos in each phenotypic category (Grade 0–3) for each treatment group. Numbers inside parentheses denote numbers of animals analyzed for each treatment group. Asterisks and brackets represent *p*<0.05 by Mann-Whitney U test.

To inhibit Dcc function in zebrafish, we injected a morpholino antisense oligonucleotide (*dcc*-MO) that blocked Dcc protein synthesis [29]. In embryos injected with *dcc*-MO (6 ng per embryo), the expression of Dcc protein was reduced to levels below the detection limits of our Western blot analyses (Fig. 3A). Photo-conversion of Kaede in *dcc*-MO injected *lhx5:Kaede* animals revealed that ADt neurons sent long processes dorsally, in addition to the normal ventrally projecting axons (Fig. 3B). These dorsally projecting processes sometimes occupied lateral positions and extended posteriorly, or they crossed the midline and extended into the contralateral side of the telencephalon (Fig. 3B). Injection of *dcc*-MO did not affect ADt neuron migration from the medial region to the lateral region of the telencephalon. The dorsally projecting processes were rarely observed in animals injected with a standard control morpholino. To summarize and compare our results, we analyzed each confocal data file and quantified the pixel intensity values of aberrant dorsal processes using custom written ImageJ macro and MATLAB scripts (Fig. S1). To present the quantified results, we assigned phenotypic scores (Grade 0–3) to each embryo based on the pixel intensity value (Fig. S1). Embryos with more pronounced dorsal processes were assigned higher scores (Fig. 3C). Average phenotypic scores (a.p.s.) were calculated for each treatment group where a higher a.p.s. reflected more severe aberrant dorsal projections. For statistical analyses, we used the non-parametric Mann-Whitney U method to test whether samples of two independent observations had equally severe phenotypic values. The rank based Mann-Whitney U test had the advantage that the statistical conclusions were independent of the specific phenotypic score matrix we employed. Based on this analysis, the *dcc*-MO injected embryos (a.p.s = 0.983) were significantly different from the standard control morpholino injected animals (a.p.s = 0.063) (Fig. 3C, *p* = 7.01×10^−7^). Similar statistical results were obtained using the original quantification data and the ANOVA method (Fig. S1). Injection with a lower dosage of *dcc*-MO (3 ng per embryo) reduced the severity (a.p.s. = 0.500) of the aberrant dorsal axons. Injection with a higher dosage of *dcc*-MO (12 ng per embryo) caused early death in about 30% of the injected embryos (52 out of 156). The intensity of the abnormal dorsal axon was increased in the remaining embryos, but the differences were not statistically significant (a.p.s = 1.13, *p* = 0.425 versus embryos injected with 6 ng of *dcc*-MO). Embryos were injected with 6 ng of *dcc*-MO in following experiments.

We used two methods to validate the specificity of the *dcc*-MO knockdowns. We examined phenotypes caused by injection of a mismatch control morpholino, and we tested for rescue of the *dcc* knockdown phenotypes by coinjection of a *dcc*-MO resistant mRNA (*dcc*-R-mRNA, Fig. 3C). We designed a mismatch control morpholino that contained 5 mismatch bases. In embryos injected with the mismatch control morpholino at the same dose as the *dcc*-MO, we rarely observed dorsally projecting processes (a.p.s. = 0.065, *p* = 1.31×10^−6^ versus *dcc*-MO injected group). To construct the *dcc* mRNA whose translation was resistant to the blocking effects of the *dcc*-MO, we introduced 7 silent mutations into the *dcc* mRNA sequence. After co-injection of the *dcc*-MO and the *dcc* mRNA with silent mutations, the occurrence and severity of the dorsal processes were significantly reduced (a.p.s. = 0.281, *p* = 4.62×10^−4^ versus *dcc*-MO injected embryos). Similar statistical results were obtained using the original quantification data and the ANOVA method (Fig. S1). These results confirmed that aberrant ADt axon formation was due to specific inhibition of the function of Dcc.

### Knocking down Dcc Function Causes ADt Neurons to Project Axons Dorsally or to form Multiple Processes

**Figure 4:**
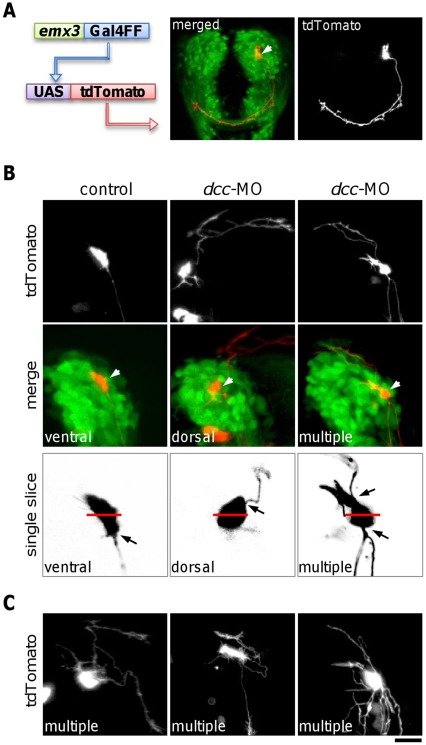
Inhibition of Dcc function causes ADt axons to project dorsally or to form multiple processes. (**A**) Labeling of individual ADt neurons by mosaic expression of fluorescent protein tdTomato. Image of a live 36 hpf *Tg(lhx5BAC:Kaede)* animal that was injected with *emx3:Gal4FF* and *UAS:tdTomato* plasmids is shown. The tdTomato labeled neuron projected an axon into the AC. Merge panel shows the position of the tdTomato labeled soma (marked by an arrowhead). Scale bar = 50 µm. (**B**) Injection of *dcc* morpholino causes ADt neurons to project axons dorsally or to form multiple processes. Labeled ADt neurons are marked by arrowheads in the merged panels. Left panels show an ADt neuron with a normal ventrally projecting axon in a control animal. Middle panels show an ADt neuron with an aberrant dorsally projecting axon in a *dcc*-MO injected animal. Right panels show an ADt neuron with both ventrally and dorsally projecting processes. Black arrow in the single slice images indicates the origin of the axon on the surface of the cell body. Red bar indicates the middle of the dorsal and the ventral side of the labeled neuron cell body. Scale bar equals to 20 µm in the projected images or 10 µm in the single slice images. (**C**) Additional examples of ADt neurons with multiple aberrant axons in *dcc*-MO injected animals. Scale bar = 15 µm.

Because our Kaede photo-conversion results could not discriminate whether the ADt neurons extended single aberrant or multiple processes in the *dcc*-MO injected embryos, we labeled single ADt neurons with a transient transgenic method (Fig. 4A). Plasmids injected into early stage zebrafish embryos are mosaically distributed among the dividing cells, and mosaic expression of transgenes encoded by the injected plasmids may result in random labeling of single or small numbers of cells in the injected embryos [30], [31]. We coinjected a plasmid carrying *emx3:Gal4FF* and a plasmid carrying *UAS:tdTomato*, with and without *dcc*-MO into *lhx5:Kaede* embryos at the one cell stage. The *emx3:Gal4FF* plasmid carries a Gal4 transcriptional activator variant [32] under control of a forebrain specific enhancer element from the *emx3* gene [33]. The *UAS:tdTomato* plasmid carries *tdTomato*, a red fluorescent protein gene [34] under control of 5 copies of the Gal4 recognition element UAS. By screening the injected embryos for axons that can be traced and assigned to single labeled ADt neurons, we obtained ADt neuronal projection patterns resolvable at single cell resolution (Fig. 4B). Among the labeled ADt neurons in control animals, none had dorsally projecting axons (Fig. 4B, n = 80). In contrast, about 40% (19 out of 50) of the labeled ADt neurons in *dcc*-MO injected animals had either dorsally projecting axons or multiple processes (Fig. 4B). The dorsally projecting axons extended laterally or crossed the midline to extend into the contralateral telencephalon (n = 7). The axons with multiple processes often projected both dorsally and ventrally (n = 12). We further examined all single confocal slices to determine the origins of the axons on the surface of the labeled neurons (Fig. 4B). In control embryos, the origins of the axons were found overwhelmingly on the ventral side of the cell bodies (92%, 74 out of 80). The other 6 labeled neurons either had the axon origins on the dorsal side (4%, n = 3) or close to the middle (4%, n = 3) of the cell bodies. In contrast, for those labeled neurons that had dorsally projecting axons in *dcc*-MO injected embryos, the origins of the axons were found on the dorsal side of the cell bodies (84%, 16 out of 19). Thus, the single cell labeling results mirrored our results obtained from group labeling, and together these results suggest that Dcc function is required for the asymmetric outgrowth of the ADt axons. Furthermore, because the labeled ADt neurons always extend single axons in control animals, the formation of ADt neuron axons with multiple processes in *dcc* morpholino injected embryos (Figure 4C) suggest that Dcc function is required for polarized axon initiation of the forebrain neurons *in vivo*.

### Inhibition of Netrin1 Function Affects the Asymmetric Growth of the ADt Neurons

**Figure 5:**
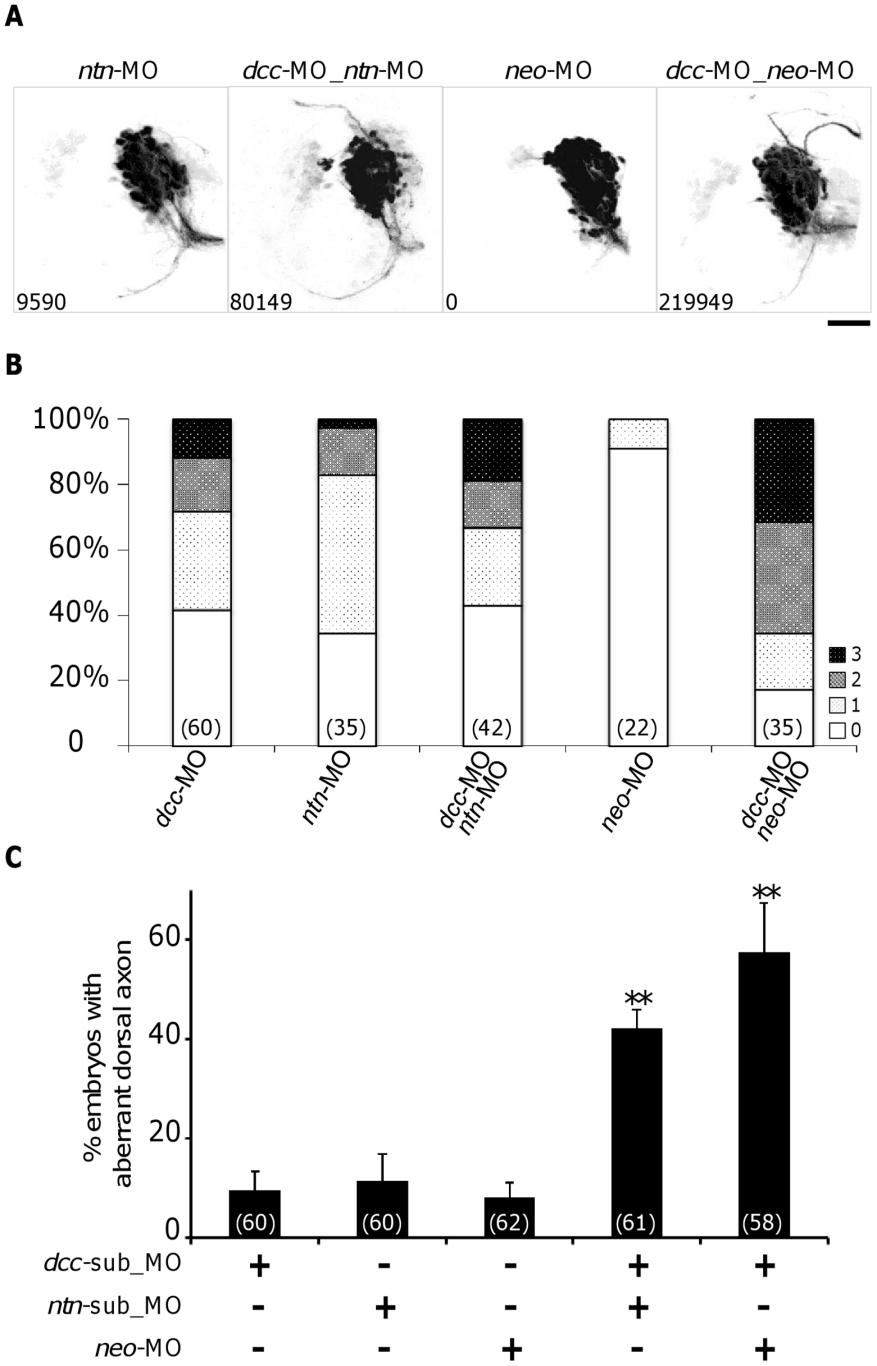
Effects of inhibition of Netrin1 or Neogenin1 function on the ADt axons. (**A**) ADt neurons project axons dorsally when Netrin1 function is inhibited (*ntn*-MO). Knockdown of Neogenin1 function doesn’t cause ADt neuron to project axon dorsally (*neo*-MO). Images were processed as in Fig. 3B. The pixel intensity value of aberrant axon is shown in the bottom left corner of each panel. Scale bar = 50 µm. (**B**) Quantitation of ADt neuronal axon defects. Horizontal axis shows the treatment group labels and vertical axis shows the percentage of embryos in each phenotypic category (Grade 0–3) for each treatment group. Numbers inside parentheses denote numbers of animals analyzed for each treatment group. (**C**) Synergistic effects between sub-threshold Dcc-Netrin1 and Dcc-Neogenin morpholino knockdowns. *dcc*-sub_MO and *ntn*-sub_MO: sub-threshold concentration morpholino. At least three independent injections were performed for each treatment group. Numbers inside parentheses denote numbers of animals analyzed. Asterisks represent *p*<0.001 by ANOVA test.

To provide further evidence for the role of Dcc in the asymmetric growth of ADt axons, we examined the effect of knocking down Netrin1, a Dcc ligand. In *lhx5:Kaede* embryos co-injected with *ntn1a* and *ntn1b* morpholinos (referred to as *ntn1*-MO, 6 ng each per embryo), ADt neurons developed aberrant dorsal projections similar to neurons in *dcc*-MO injected embryos (Fig. 5A, a.p.s. = 0.857, *p* = 0.820 versus *dcc*-MO injected embryos). Injection of either *ntn1a*-MO or *ntn1b*-MO alone didn’t cause ADt neurons to project axons dorsally. Co-injection of *ntn1a* and *ntn1b* mismatch control morpholinos didn’t cause ADt neurons to develop aberrant projections (data not shown). Knocking down both Dcc and Netrin1 by co-injecting *dcc*-MO (6 ng per embryo) and *ntn1*-MO (3 ng each per embryos) caused the ADt neurons to develop aberrant projections in a manner similar to knock down of Dcc alone (a.p.s. = 1.09, Fig. 5B and Fig. S2). A lower dose of *ntn1*-MO (3 ng each versus 6 ng each per embryo) was used in the Dcc-Netrin1 double knockdown experiment due to concerns over potential toxicity of high dose morpholino injections.

To test the genetic interaction between the Dcc and Netrin1 knockdowns further, we examined the synergistic effect of Dcc-Netrin1 morpholino injections at sub-threshold concentrations that did not cause significant aberrant axon phenotype when injected alone [35], [36]. We first generated dose response curves for the Dcc and Netrin1 single knockdowns (Fig. S3). When a sub-threshold dose of *dcc*-MO (1.5 ng per embryo) was injected, 9.6±3.7% of the injected embryos developed dorsal axons (n = 60). Injection of a sub-threshold dose of *ntn*-MO (1.5 ng each per embryo) caused aberrant dorsal axons in 11.5±5.4% of embryos (n = 60). These penetrance numbers were not significantly different from the percentage of aberrant axons observed in standard control morpholino injected embryos (9.0±6.4%, n = 55; ANOVA, *F*_2, 7_ = 0.185, *p* = 0.835). On the other hand, co-injection of the sub-threshold concentrations of *dcc*-MO and *ntn*-MO together resulted in the formation of significant number of aberrant dorsal axons (42.1±3.7% of embryos, n = 61; ANOVA, *F*_2, 6_ = 52.31, *p* = 1.60×10^−4^, Fig. 5C). These results indicated that Dcc and Netrin1 genetically interacted to determine the asymmetric outgrowth of the ADt neuronal axons.

### Neogenin1 Knockdown Enhances the Aberrant ADt Axons in *dcc*-MO Injected Embryos

The guidance receptor Neogenin is closely related to DCC [37], [38]. Neogenin1 is expressed in the central nervous system and the mesoderm throughout early development in zebrafish embryos [39], [40]. Previous studies showed that knock down of Neogenin1 function by morpholino injection caused abnormal development of the somite and the central nervous system in zebrafish embryos [40], [41]. We examined whether inhibition of Neogenin1 function also affected the asymmetric growth of ADt neurons. In embryos injected with *neogenin1* morpholino (*neo*-ATGMO, 2 ng per embryos [40]), the aberrant dorsal ADt neuronal projections (a.p.s. = 0.091) were rarely observed and were not significantly different from those in the standard control morpholino injected embryos (*p* = 1.000). Injection of higher dosages of *neo*-ATGMO (4 ng per embryos) caused reductions of the AC or both the AC and the SOT (Fig. 5A), whilst it didn’t result in significant aberrant dorsal projections (a.p.s. = 0.053, *p* = 1.000 versus standard control morpholino injected embryos). Similar to previous report [40], increasing the dosage of the *neo*-ATGMO further (8 ng per embryo) caused malformation of the neural tube and the ADt neurons (data not shown). When we co-injected both the *neo*-ATGMO (2 ng per embryo) and the *dcc*-MO (6 ng per embryo) into the *lhx5*:Kaede embryos (Fig. 5A), the aberrant ADt neuronal projections were enhanced (Fig. 5B, a.p.s. = 1.800, Fig. 5B and Fig. S2). Co-injection of the sub-threshold *dcc*-MO (1.5 ng per embryo) and the *neo*-MO (2 ng per embryo) caused a significant increase in the number of aberrant dorsal axons (57.5±9.9%, n = 58) comparing with the single injections (8.1±3.0%, n = 62, in *neo*-MO injected embryos; ANOVA, *F*_2, 6_ = 58.49, *p* = 1.16×10^−4^, Fig. 5C). These result indicated that Neogenin1 may partially compensate for the loss of the Dcc function in the *dcc*-MO injected embryos.

## Discussion

### DCC Regulates Polarized Initiation and Asymmetric Outgrowth of Forebrain Neuronal Axons

Our studies demonstrate that Dcc signaling plays a central role in asymmetric outgrowth of ADt neuronal axons in the zebrafish forebrain. Dcc and its ligand, Netrin1, are expressed prior to the time that ADt neurons extend their axons ventrally (Fig. 2). Knock down of Dcc function caused ADt axons to extend aberrantly (Fig. 3). These incorrect axonal projections are not likely due to abnormal specification of the ADt neurons, because the neurons migrated to their correct locations and differentiated along their normal time course. Single cell labeling showed that ADt neurons projected axons dorsally or formed multiple axons (Fig. 4). Because the labeled ADt neurons always extend single axons in control animals, the formation of ADt neuron axons with multiple processes in *dcc* morpholino injected embryos suggests that Dcc signaling is required for polarized axon initiation of the forebrain neurons *in vivo*. Previous studies in *C. elegans* showed that the correct asymmetry of HSN neurons was lost in *unc-40* (*dcc*) mutants [11] and HSN axons deviated from their correct ventral trajectory to extend dorsally [15]. Thus, our results in zebrafish mirror those observed in *C. elegans*, suggesting that the functions of Dcc in asymmetric outgrowth of neurons are likely conserved in metazoans.

Previous studies have shown that DCC signaling may promote axonal branching in cultured cortical pyramidal neurons [42], and that disrupting DCC function leads to a decrease in the number of axon terminals in cultured dopaminergic neurons [43]. In *Dcc* knockout mice, ventral mitral cell axons deviated from their normal path and extended towards more dorsal locations [44]. A recent study showed that cultured hippocampal neurons developed multiple axons when DCC expression was reduced in the context of Bcl11A knockdown [45]. These observations, together with our results, suggest that DCC’s function in neuronal polarity regulation may depend on the particular environment experienced by developing neurons.

Our studies show that DCC, Netrin1, and Neogenin1 play overlapping and differential roles in the asymmetric outgrowth of ADt neuronal axons. Inhibition of the function of either Dcc or Netrin1 caused ADt neuron to project axons dorsally, whereas inhibition of Neogenin function didn’t result in significant dorsal ADt axons (Fig. 5). These results suggest that Netrin1-Dcc signaling plays a primary role in directing the asymmetric outgrowth of forebrain neuronal axons. Interestingly, knock down of Dcc and Neogenin1 together enhanced the formation of aberrant dorsal ADt axon. This result suggests that Neogenin1 may act in parallel with Dcc and that Neogenin may partially compensate for loss of Dcc function in *dcc*-MO injected embryos. Because knock down of Dcc and Neogenin1 together caused more aberrant dorsal ADt axons than knock down of Dcc and Netrin1 together, it is likely that other cues in addition to Netrin1 mediate asymmetric outgrowth of the ADt axons. Netrin2 and Netrin4, two Netrin family member proteins that may interact with Dcc in zebrafish, are not expressed in the forebrain prior to 36 hpf [46] and thus are unlikely to act with Netrin1 as ADt guidance factors. On the other hand, RGMa, Draxin and MADD-4 (ADAMTL1 and ADAMTL3) are known to interact genetically or physically with Dcc or Neogenin1 [41], [47]–[49]. Further studies are needed to determine whether these additional factors are involved in the asymmetric outgrowth of the ADt neurons.

### Transgenic Labeling of Forebrain Axons

Few previous studies have examined the roles of DCC in neuronal polarity regulation in vertebrates. We took advantage of transgenic labeling methods to investigate function of Dcc in ADt axon development in zebrafish. The Kaede photo-conversion method allowed specific labeling of ADt axons. Such specific labeling should facilitate future studies of gain and loss of function of other genes that may be involved in ADt axon development. Due to diffraction and scattering of the laser beam in live intact animals, other photo-convertible fluorescent proteins such as KikGR [50], in combination with two-photon conversion may be required to study cell population located in deeper regions.

Our study also utilized single cell labeling by generating mosaic transgenic animals. Single cell labeling provides increased specificity and should be useful in studies of cell migration and neural circuit formation. The mosaic labeling method requires injection and screening of many animals; in our studies, fewer than 10% of the injected embryos yielded useful information and screening by confocal microscopy was time consuming. Although such analyses are much more efficient in C. *elegans*, where stereotyped neurons can be observed routinely at single cell resolution, further analyses of single cells in vertebrate nervous systems are warranted due to the large numbers of different cells types and neuronal connections.

## Materials and Methods

### Fish Maintenance

Zebrafish were maintained in a recirculating water system according to standard protocol [51]. Recirculated water was dosed to 500µF salinity with artificial ocean salt mix and buffered to pH7.2 with NaHCO_3_. Embryos were obtained by breeding adult fish and raised at 28.5°C as described [51]. Embryos were staged by hours post fertilization (hpf) as described [52]. The Fudan University Institutional Animal Care and Use Committee approved all work with zebrafish animals.

### Cloning

Zebrafish *dcc* cDNAs were amplified by RT-PCR with primers *dcc*-F1 (5′-CTCCACCATGG GCTGCGTCACTGGAG), *dcc*-R1 (5′-GATGAGCGCCACAATCAGCACCACCAC), *dcc*-F2 (5′- GGGGTCTCGGATAAAGACACACTCATCACC), *dcc*-R2 (5′-GTAAACGCTGATCCT GTGATGGCGTTG). The two overlapping *dcc* cDNA fragments were then assembled into a full length *dcc* cDNA clone. To construct a *dcc* cDNA that was resistant to anti-sense blocking effects (*dcc-*R-mRNA), silent mutations were introduced into the *dcc* cDNA sequence with forward primer *dcc*-MO-resistant (5′-CTCAAGCTTCGAATTCTCCACCATGGGGTGTG TGACAG GTGACATACGCCGACTTTCCGCGCTCCTC, the mutated nucleotides are underlined).

### Synthetic mRNA and Morpholinos

Capped mRNA was synthesized with mMESSAGE mMACHINE kit (Ambion). The dosage for mRNA injections was 350 pg of *dcc*-R-mRNA per embryo.

Morpholinos were synthesized by Gene Tools: standard control: 5′-CCTCTTACCTCAGTT ACAATTTATA; *dcc*-MO: 5′-GAATATCTCCAGTGACGCAGCCCAT [29]; *dcc*-misMO: 5′-CAATATGTCCACTGACCCAGCGCAT (mismatch nucleotides underlined); *ntn1a*-MO: 5′- CCAAAGCATCAGAGACTCTCAACAT; *ntn1b*-MO: 5′- CGCACGTTACCAAAATCCTT ATCAT [29]; *ntn1a*-misMO: 5′- CGAAACCATCACAGACTGTCAAGAT; *ntn1b*-misMO: 5′- CGGACCTTACGAAAATGCTTATGAT; *neo*-ATGMO: GGCTCCCCGCTCCGCCATCACT TTA [40]. The dosages for morpholino injections were: 6 ng of standard control, *dcc*-MO, *dcc*-misMO, *ntn1a*-MO, *ntn1b*-MO, *ntn1a*-misMO, *ntn1b*-misMO, and 2 ng of *neo*-ATGMO per embryo. *dcc*-MO, *ntn*-MO and *neo*-ATGMO were also injected at various doses as indicated in the result section.

### Generation of *Tg(lhx5BAC:Kaede)* Transgenic Line

A zebrafish bacteria artificial chromosome clone (BAC) containing the *lhx5* coding regions (cloned id: CH211-230H6) was identified using a genomic database search (http://www.ensembl.org/Danio_rerio/Info/Index) and fingerprinting analysis (http://www.sanger.ac.uk/Projects/D_rerio/WebFPC/zebrafish/large.shtml). Targeted modification of the BAC with the photoconvertible fluorescent protein Kaede [22] was based on homologous recombination [23], and microinjection of BAC DNA was previously described [24]. To generate the targeting fragments for homologous recombination, plasmid DNA containing Kaede and a kanamycin resistant cassette flanked by FRT-sites were used as templates with oligonucleotide primers *kaede*-F (5′- *CCCAATCCGAGGCGCGGTGCCCGTTTGGGAAGGTCG GCTCTCGGTCCCC TCCAGGGCGGA* CACCATGAGTCTGATTAAACCAG ) and *kaede*-R (5′- *GAGGATTAGTCCAGAAGTTTGACGAGGTGATGCAGCCAGAATGCAAGCAGCTTCGC GTCTT* CTATTCCA GAAGTAGTGAGGAG ). For these primers, nucleotides in italics are homologous to the targeted *lhx5* gene, and those underlined are homologous to the plasmid DNA templates. The kanamycin resistant marker was removed by induction of Flpe expression [23]. Homologous insertion of the transgenes and the removal of the kanamycin marker were verified by PCR analysis. Integrity of the modified BAC was checked by fingerprinting analysis. The *Tg(lhx5BAC:Kaede)* line was maintained by outcross and incross protocols in our fish facility.

### Whole-mount in situ Hybridization

Whole-mount *in situ* hybridization was performed as described [21]. Digoxigenin-labeled antisense RNA probes were hybridized and then detected with alkaline phosphatase-conjugated digoxigenin antibody Fab fragment (1∶7500, Roche) and alkaline phosphatase substrate NBT/BCIP (1∶80, Roche). Fluorescent *in situ* hybridization was performed as described [53]. Digoxigenin-labeled *dcc* and fluorescein-labeled *lhx5* antisense RNA probes were detected by Tyramide Signal Amplification kits (Invitrogen). The clones used in this study are: *lhx5* [21], *dcc* [29], *ntn1a* (accession number: BC114259), and *ntn1b* (accession number: CK684895). All clones were verified by sequencing.

### Photo-conversion of Kaede

Photo-conversion of Kaede was performed with a confocal microscope (Olympus FV1000) equipped with SIM module and 405 nm laser. Live embryos were mounted with 1.2% low melting temperature Agarose and the ADt region on one hemisphere of the forebrain was marked with the polygon selection tool of the FluoView software (Olympus). Ten pulses of focused 405 nm laser light were then applied to the selected region. Each pulse lasted 500 msec and utilized 60% of the light output of the 405 nm laser. Confocal image stacks were acquired 30 min later after the converted Kaede diffused into the neuronal processes.

### Western Blot

Embryos were deyolked in cold Ringer’s solution supplemented with 1 mM EDTA, 0.3 mM PMSF and protease inhibitor cocktail (Roche). Deyolked embryos were lysed in SDS-sample buffer and heated at 95°C to extract and denature protein. Protein samples were resolved on SDS-PAGE gel then transferred to nitrocellulose membrane (Whatman). Rabbit zebrafish Dcc antibodies (AnaSpec) were used at 1∶50. The specificity of the Dcc antibodies were verified by cross checking with antibodies against Myc (AbMart) and GFP (Invitrogen) for heterologous expression of Myc- and GFP-tagged Dcc. Endogenous Dcc protein was detected as a band of approximately 170 kb.

### Genetic Single Cell Labeling

A mixture of two plasmids *−5.2emx3:Gal4FF* and *5*x*UAS:tdTomato* (10 ng/µl each) was injected into one-cell stage embryos. Injected embryos were screened by fluorescent dissection microscopy at 24 hpf and then by confocal microscopy at 36 hpf to identify animals with axons that can be traced and assigned to labeled single cells. To label cells in *dcc* morpholino injected animals, *dcc* morpholino was injected at one- to four-cell stage after the plasmid injection.

### Microscopy and Image Analysis

Whole mount *in situ* images were acquired on a dissection microscope with a CCD camera (Leica M205FA). For confocal microscopy, live or fixed embryos were mounted with 1.2% low melting temperature Agarose and imaged on an Olympus FV1000 confocal laser scanning system with 40x water immersion objective. The captured image stacks were converted by ImageJ (http://rsbweb.nih.gov/ij/) for reconstruction with FluoRender software (http://www.sci.utah.edu/software/46-documentation/137-fluorender.html). Reconstructed three dimensional images were projected to a standardized view for figure presentation. To quantify the aberrant projection phenotypes, confocal data files were automatically processed by custom written ImageJ macro and MATLAB scripts (Fig. S1, Text S1, and Text S2). For statistical analysis, non-parametric Mann-Whitney U tests and ANOVA tests were performed with SPSS software. Two-tailed exact *p* values were reported for the Mann-Whitney U tests and significance *P* values were reported for the ANOVA tests (SPSS Inc.).

## Supporting Information

Figure S1 **Quantification of aberrant dorsal axons.** (**A**) Procedure of quantification. The ImageJ marco extracts image stacks, selects the converted Kaede channel, uses maximum intensity method to project the image stacks along the Z axis, and saves the projections as gray scaled images. The processed images were manually cropped to rectangles containing aberrant axons and the cell bodies were masked with GIMP software. The MATLAB scripts use an adaptive thresholding method to index the axon images then calculate the sum of the pixel intensity values corresponding to the indexed axonal positions. One example of the indexed axon image and its pixel intensity value are shown below the rendered confocal image. The indexed axon positions are in white. (**B**) Quantification and statistics test results. Increased shading corresponds to Grade 0–3. Grade 0: pixel intensity value less than 5000; Grade 1: less than 25000; Grade 2: less than 45000; Grade 3: greater than 45000. Statistic test results (*F* and *P* values) with the quantification data and the ANOVA methods are given at the bottom. Post Hoc test results between *dcc*-MO injected and other experimental conditions are given on the header lines.
(TIFF)

Figure S2 **Pixel intensity values of aberrant axons in** ***ntn*** **-MO and** ***neo*** **-MO injected embryos.** Quantification results are shaded as in Fig. S1.
(TIF)

Figure S3 **Dose response curves of Dcc and Netrin1 morpholino injections.** 2 nl of *dcc*-MO or *ntn*-MO morpholino stocks diluted to appropriate concentrations were injected into the 1- to 2-cell embryos. The number of embryos analyzed were 60 (1.5 ng), 48 (3 ng), 60 (6 ng), and 23 (9 ng) for *dcc*-MO; 60 (1.5 ng each), 43(3 ng each), and 35 (6 ng each) for *ntn*-MO.
(TIFF)

Text S1 ImageJ macro used in quantification of aberrant dorsal axons.
(TXT)

Text S2 MATLAB scripts used in quantification of aberrant dorsal axons.
(M) Click here for additional data file.

## References

[1] Dotti CG, Sullivan CA, Banker GA (1988). The establishment of polarity by hippocampal neurons in culture.. J Neurosci.

[2] Shi S-H, Jan LY, Jan Y-N (2003). Hippocampal neuronal polarity specified by spatially localized mPar3/mPar6 and PI 3-kinase activity.. Cell.

[3] Jiang H, Guo W, Liang X, Rao Y (2005). Both the establishment and the maintenance of neuronal polarity require active mechanisms: critical roles of GSK-3beta and its upstream regulators.. Cell.

[4] Yoshimura T, Kawano Y, Arimura N, Kawabata S, Kikuchi A (2005). GSK-3beta regulates phosphorylation of CRMP-2 and neuronal polarity.. Cell.

[5] Kwiatkowski AV, Rubinson DA, Dent EW, Edward van Veen J, Leslie JD (2007). Ena/VASP Is Required for neuritogenesis in the developing cortex.. Neuron.

[6] Barnes AP, Lilley BN, Pan YA, Plummer LJ, Powell AW (2007). LKB1 and SAD kinases define a pathway required for the polarization of cortical neurons.. Cell.

[7] Shelly M, Cancedda L, Heilshorn S, Sumbre G, Poo M-M (2007). LKB1/STRAD promotes axon initiation during neuronal polarization.. Cell.

[8] Arimura N, Kaibuchi K (2007). Neuronal polarity: from extracellular signals to intracellular mechanisms.. Nat Rev Neurosci.

[9] Zolessi FR, Poggi L, Wilkinson CJ, Chien C-B, Harris WA (2006). Polarization and orientation of retinal ganglion cells in vivo.. Neural Devlop.

[10] Hilliard MA, Bargmann CI (2006). Wnt signals and frizzled activity orient anterior-posterior axon outgrowth in C. elegans.. Dev Cell.

[11] Adler CE, Fetter RD, Bargmann CI (2006). UNC-6/Netrin induces neuronal asymmetry and defines the site of axon formation.. Nat Neurosci.

[12] Prasad BC, Clark SG (2006). Wnt signaling establishes anteroposterior neuronal polarity and requires retromer in C. elegans.. Development (Cambridge, England).

[13] Yi JJ, Barnes AP, Hand R, Polleux F, Ehlers MD (2010). TGF-beta signaling specifies axons during brain development.. Cell.

[14] Barnes AP, Polleux F (2009). Establishment of axon-dendrite polarity in developing neurons.. Annu Rev Neurosci.

[15] Xu Z, Li H, Wadsworth WG (2009). The roles of multiple UNC-40 (DCC) receptor-mediated signals in determining neuronal asymmetry induced by the UNC-6 (netrin) ligand.. Genetics.

[16] Hedgecock EM, Culotti JG, Hall DH (1990). The unc-5, unc-6, and unc-40 genes guide circumferential migrations of pioneer axons and mesodermal cells on the epidermis in C. elegans.. Neuron.

[17] Kolodziej PA, Timpe LC, Mitchell KJ, Fried SR, Goodman CS (1996). frazzled encodes a Drosophila member of the DCC immunoglobulin subfamily and is required for CNS and motor axon guidance.. Cell.

[18] Fazeli A, Dickinson SL, Hermiston ML, Tighe RV, Steen RG (1997). Phenotype of mice lacking functional Deleted in colorectal cancer (Dcc) gene.. Nature.

[19] Yee KT, Simon HH, Tessier-Lavigne M, O’Leary DM (1999). Extension of long leading processes and neuronal migration in the mammalian brain directed by the chemoattractant netrin-1.. Neuron.

[20] Wilson SW, Ross LS, Parrett T, Easter SS (1990). The development of a simple scaffold of axon tracts in the brain of the embryonic zebrafish, Brachydanio rerio.. Development (Cambridge, England).

[21] Peng G, Westerfield M (2006). Lhx5 promotes forebrain development and activates transcription of secreted Wnt antagonists.. Development (Cambridge, England).

[22] Ando R, Hama H, Yamamoto-Hino M, Mizuno H, Miyawaki A (2002). An optical marker based on the UV-induced green-to-red photoconversion of a fluorescent protein.. Proc Natl Acad Sci USA.

[23] Lee EC, Yu D, Martinez de Velasco J, Tessarollo L, Swing DA (2001). A highly efficient Escherichia coli-based chromosome engineering system adapted for recombinogenic targeting and subcloning of BAC DNA.. Genomics.

[24] DeLaurier A, Eames BF, Blanco-Sánchez B, Peng G, He X (2010). Zebrafish sp7:EGFP: a transgenic for studying otic vesicle formation, skeletogenesis, and bone regeneration.. Genesis (New York, N Y : 2000).

[25] Fricke C, Chien C-B (2005). Cloning of full-length zebrafish dcc and expression analysis during embryonic and early larval development.. Dev Dyn.

[26] Hjorth JT, Gad J, Cooper H, Key B (2001). A zebrafish homologue of deleted in colorectal cancer (zdcc) is expressed in the first neuronal clusters of the developing brain.. Mech Dev.

[27] Lauderdale JD, Davis NM, Kuwada JY (1997). Axon tracts correlate with netrin-1a expression in the zebrafish embryo.. Mol Cell Neurosci.

[28] Strähle U, Fischer N, Blader P (1997). Expression and regulation of a netrin homologue in the zebrafish embryo.. Mech Dev.

[29] Suli A, Mortimer N, Shepherd I, Chien C-B (2006). Netrin/DCC signaling controls contralateral dendrites of octavolateralis efferent neurons.. J Neurosci.

[30] Westerfield M, Wegner J, Jegalian BG, DeRobertis EM, Püschel AW (1992). Specific activation of mammalian Hox promoters in mosaic transgenic zebrafish.. Genes Dev.

[31] Miyasaka N, Morimoto K, Tsubokawa T, Higashijima S-ichi, Okamoto H (2009). From the olfactory bulb to higher brain centers: genetic visualization of secondary olfactory pathways in zebrafish.. J Neurosci.

[32] Asakawa K, Suster ML, Mizusawa K, Nagayoshi S, Kotani T (2008). Genetic dissection of neural circuits by Tol2 transposon-mediated Gal4 gene and enhancer trapping in zebrafish.. Proc Natl Acad Sci USA.

[33] Viktorin G, Chiuchitu C, Rissler M, Varga ZM, Westerfield M (2009). Emx3 is required for the differentiation of dorsal telencephalic neurons.. Dev Dyn.

[34] Shaner NC, Campbell RE, Steinbach PA, Giepmans BNG, Palmer AE (2004). Improved monomeric red, orange and yellow fluorescent proteins derived from Discosoma sp. red fluorescent protein.. Nat Biotech.

[35] Maves L, Jackman W, Kimmel CB (2002). FGF3 and FGF8 mediate a rhombomere 4 signaling activity in the zebrafish hindbrain.. Development (Cambridge, England).

[36] Feldner J, Reimer MM, Schweitzer J, Wendik B, Meyer D (2007). PlexinA3 restricts spinal exit points and branching of trunk motor nerves in embryonic zebrafish.. J Neurosci.

[37] De Vries M, Cooper HM (2008). Emerging roles for neogenin and its ligands in CNS development.. J Neurochem.

[38] Wilson NH, Key B (2007). Neogenin: one receptor, many functions.. Int J Biochem Cell Biol.

[39] Shen H, Illges H, Reuter A, Stuermer CAO (2002). Cloning, expression, and alternative splicing of neogenin1 in zebrafish.. Mech Dev.

[40] Mawdsley DJ, Cooper HM, Hogan BM, Cody SH, Lieschke GJ (2004). The Netrin receptor Neogenin is required for neural tube formation and somitogenesis in zebrafish.. Dev Biol.

[41] Kee N, Wilson N, De Vries M, Bradford D, Key B (2008). Neogenin and RGMa control neural tube closure and neuroepithelial morphology by regulating cell polarity.. J Neurosci.

[42] Dent EW, Barnes AM, Tang F, Kalil K (2004). Netrin-1 and semaphorin 3A promote or inhibit cortical axon branching, respectively, by reorganization of the cytoskeleton.. J Neurosci.

[43] Xu B, Goldman JS, Rymar VV, Forget C, Lo PS (2010). Critical roles for the netrin receptor deleted in colorectal cancer in dopaminergic neuronal precursor migration, axon guidance, and axon arborization.. Neuroscience.

[44] Kawasaki T, Ito K, Hirata T (2006). Netrin 1 regulates ventral tangential migration of guidepost neurons in the lateral olfactory tract.. Development (Cambridge, England).

[45] Kuo T-Y, Hong C-J, Hsueh Y-P (2009). Bcl11A/CTIP1 regulates expression of DCC and MAP1b in control of axon branching and dendrite outgrowth.. Mol Cell Neurosci.

[46] Park KW, Urness LD, Senchuk MM, Colvin CJ, Wythe JD (2005). Identification of new netrin family members in zebrafish: developmental expression of netrin 2 and netrin 4.. Dev Dyn.

[47] Wilson NH, Key B (2006). Neogenin interacts with RGMa and netrin-1 to guide axons within the embryonic vertebrate forebrain.. Dev Biol.

[48] Ahmed G, Shinmyo Y, Ohta K, Islam SM, Hossain M (2011). Draxin Inhibits Axonal Outgrowth through the Netrin Receptor DCC.. J Neurosci.

[49] Seetharaman A, Selman G, Puckrin R, Barbier L, Wong E (2011). MADD-4 Is a Secreted Cue Required for Midline-Oriented Guidance in Caenorhabditis elegans.. Developmental Cell.

[50] Tsutsui H, Karasawa S, Shimizu H, Nukina N, Miyawaki A (2005). Semi-rational engineering of a coral fluorescent protein into an efficient highlighter.. EMBO Rep.

[51] Westerfield M (2007). The Zebrafish Book: A guide for the laboratory use of zebrafish Danio rerio, 5th ed..

[52] Kimmel CB, Ballard WW, Kimmel SR, Ullmann B, Schilling TF (1995). Stages of embryonic development of the zebrafish.. Dev Dyn.

[53] Talbot JC, Johnson SL, Kimmel CB (2010). hand2 and Dlx genes specify dorsal, intermediate and ventral domains within zebrafish pharyngeal arches.. Development (Cambridge, England).

