# Supplementary figures and images for: Dcc Regulates Asymmetric Outgrowth of Forebrain Neurons in Zebrafish
Source: PLoS One. 2012 May 14;7(5):e36516. doi: 10.1371/journal.pone.0036516 (PMC3351449; doi:10.1371/journal.pone.0036516)

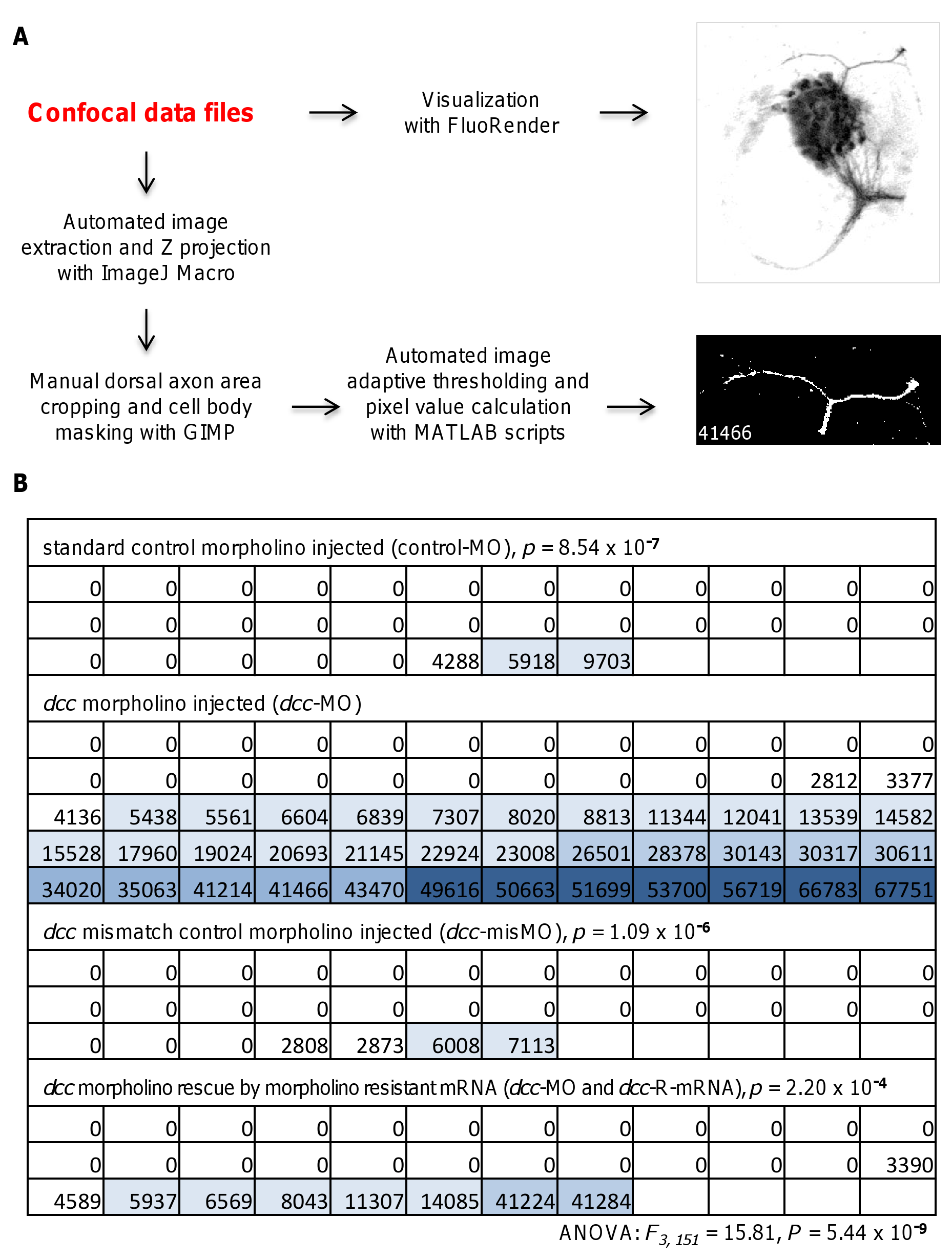

Supplement: Figure S1 — Quantification of aberrant dorsal axons. (A) Procedure of quantification. The ImageJ marco extracts image stacks, selects the converted Kaede channel, uses maximum intensity method to project the image stacks along the Z axis, and saves the projections as gray scaled images. The processed images were manually cropped to rectangles containing aberrant axons and the cell bodies were masked with GIMP software. The MATLAB scripts use an adaptive thresholding method to index the axon images then calculate the sum of the pixel intensity values corresponding to the indexed axonal positions. One example of the indexed axon image and its pixel intensity value are shown below the rendered confocal image. The indexed axon positions are in white. (B) Quantification and statistics test results. Increased shading corresponds to Grade 0–3. Grade 0: pixel intensity value less than 5000; Grade 1: less than 25000; Grade 2: less than 45000; Grade 3: greater than 45000. Statistic test results (F and P values) with the quantification data and the ANOVA methods are given at the bottom. Post Hoc test results between dcc-MO injected and other experimental conditions are given on the header lines. (TIFF) [file pone.0036516.s001.tiff]

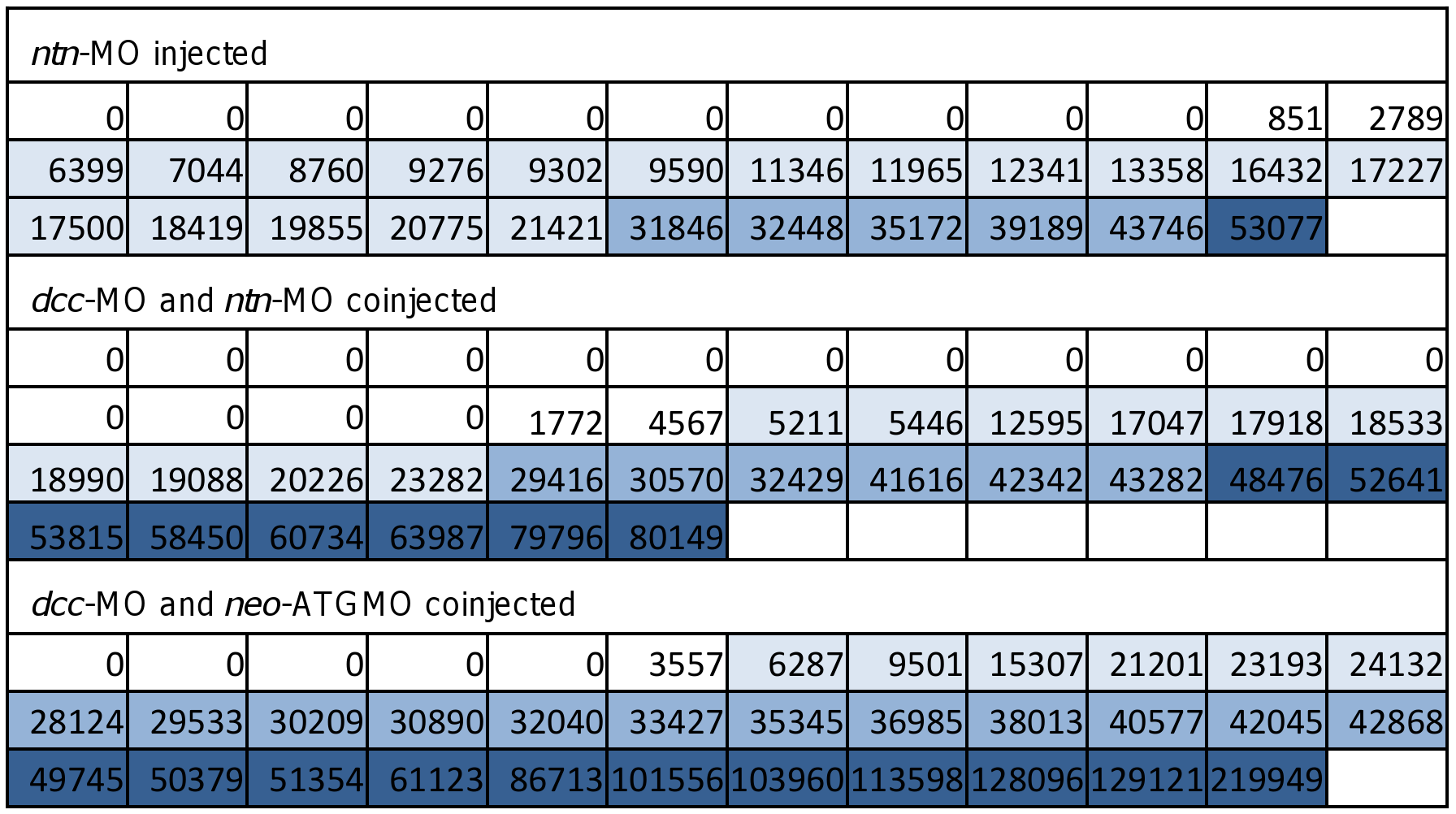

Supplement: Figure S2 — Pixel intensity values of aberrant axons in ntn -MO and neo -MO injected embryos. Quantification results are shaded as in Fig. S1. (TIF) [file pone.0036516.s002.tif]

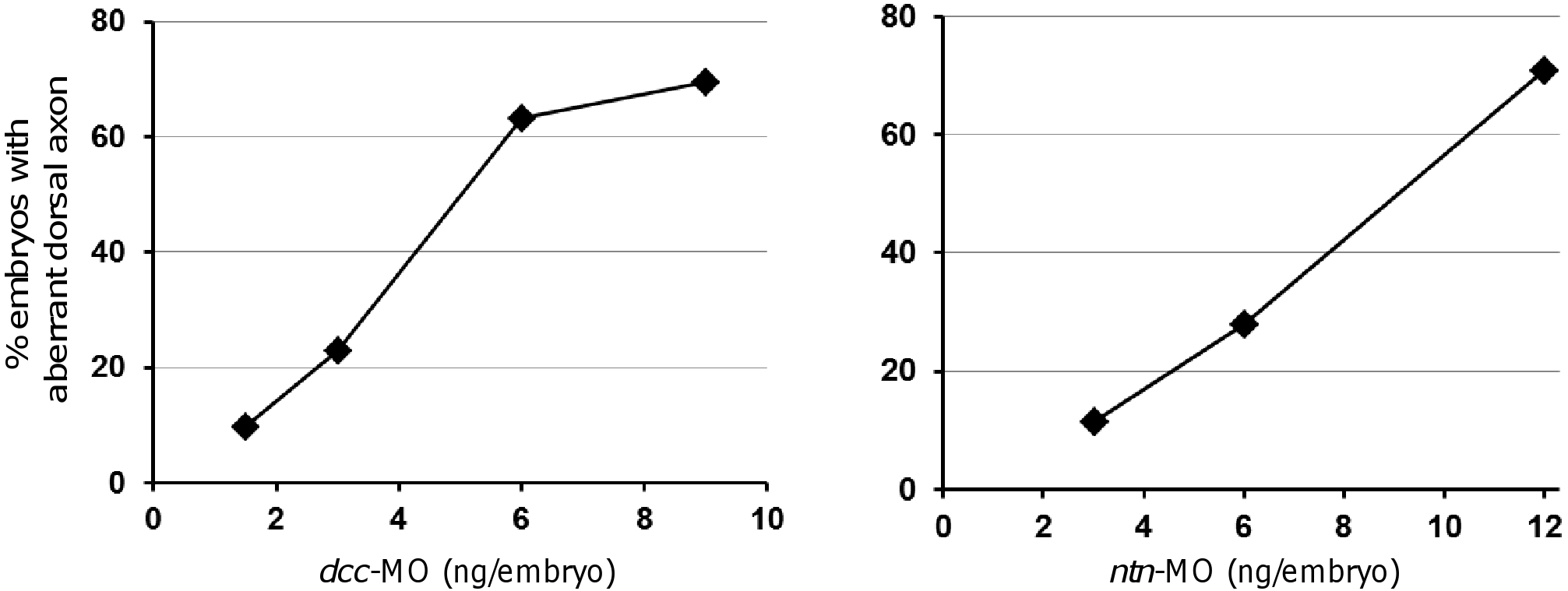

Supplement: Figure S3 — Dose response curves of Dcc and Netrin1 morpholino injections. 2 nl of dcc-MO or ntn-MO morpholino stocks diluted to appropriate concentrations were injected into the 1- to 2-cell embryos. The number of embryos analyzed were 60 (1.5 ng), 48 (3 ng), 60 (6 ng), and 23 (9 ng) for dcc-MO; 60 (1.5 ng each), 43(3 ng each), and 35 (6 ng each) for ntn-MO. (TIFF) [file pone.0036516.s003.tiff]
